# Extract of a Letter to the Editors

**Published:** 1814-12

**Authors:** 


*For the Medical and Physical Journal.*

### *Extract of a Letter to the Editors.*

I WAS much gratified by the perusal of your observations on Mr. Abernethy's Lectures. I consider it a misfortune that some men of high character should so often as they do amuse the world with theories and hypotheses, *which must be unintelligible even to themselves*, using terms either without meaning, or at least in a vague and indefinite sense, only calculated to check further investigation, and confound students who have little discernment! Mr. A. should read Locke on the Abuse of Words, and Boyle on the term "*Nature.*" ANON.

